# Adaptive periodicity in the infectivity of malaria gametocytes to mosquitoes

**DOI:** 10.1098/rspb.2018.1876

**Published:** 2018-10-03

**Authors:** Petra Schneider, Samuel S. C. Rund, Natasha L. Smith, Kimberley F. Prior, Aidan J. O'Donnell, Sarah E. Reece

**Affiliations:** 1 Institute of Evolutionary Biology and Institute of Immunology and Infection Research, School of Biological Sciences, University of Edinburgh, Charlotte Auerbach Road, Edinburgh EH9 3FL, UK; 2 Department of Biological Sciences, University of Notre Dame, Notre Dame, IN, USA

**Keywords:** *Plasmodium chabaudi*, Hawking hypothesis, transmission strategy, vector, circadian rhythm, periodicity

## Abstract

Daily rhythms in behaviour, physiology and molecular processes are expected to enable organisms to appropriately schedule activities according to consequences of the daily rotation of the Earth. For parasites, this includes capitalizing on periodicity in transmission opportunities and for hosts/vectors, this may select for rhythms in immune defence. We examine rhythms in the density and infectivity of transmission forms (gametocytes) of rodent malaria parasites in the host's blood, parasite development inside mosquito vectors and potential for onwards transmission. Furthermore, we simultaneously test whether mosquitoes exhibit rhythms in susceptibility. We reveal that at night, gametocytes are twice as infective, despite being less numerous in the blood. Enhanced infectiousness at night interacts with mosquito rhythms to increase sporozoite burdens fourfold when mosquitoes feed during their rest phase. Thus, changes in mosquito biting time (owing to bed nets) may render gametocytes less infective, but this is compensated for by the greater mosquito susceptibility.

## 1. Introduction

The rotation of the Earth, every 24 h, results in exposure to environmental (abiotic) rhythms in ambient light, temperature, humidity and ultraviolet (UV) radiation. The evolution of circadian rhythms is assumed to be an adaptation to cope with the challenges of—and exploit the opportunities provided by—predictable daily environmental rhythms [1,2]. Parasites also experience diverse environmental rhythms generated by daily rhythms in their hosts (and vectors) [3–5]. Such ‘biotic’ environmental rhythms include immune responses, resource availability and transmission opportunities. For example, Hawking [6] observed that the microfilaria (transmission forms) of *Wuchereria bancrofti*, transmitted by nocturnally active mosquitoes, only migrate to the peripheral blood of the host during the evening and move back to the lungs during the day. The timing of this migration behaviour is reversed for the Pacific type of *W. bancrofti* which is transmitted by a diurnal vector species [6,7].

The ability to schedule the expression of transmission traits for the time of day that vectors are host-seeking appears to be adaptive (i.e. maximizes parasite fitness) and could benefit many species of parasites. Thus, Hawking [8] also predicted that this hypothesis explains periodicity in the cycles of asexual replication observed in many species of malaria parasites. In the blood of the vertebrate host, malaria (*Plasmodium*) species undergo successive cycles of asexual replication which result in bouts of fever every 24, 48 or 72 h (depending on the species) when parasites burst to release their progeny. The regularity of fevers is sufficiently reliable that it was used as a diagnostic symptom in the Hippocratic era, but why there appears to be a circadian basis to the duration of asexual cycles has been a mystery ever since, especially because not all malaria species are synchronous [3,9,10]. Malaria transmission forms (gametocytes) are not motile, and unlike *W. bancrofti* microfilariae, they cannot actively migrate to accumulate in the peripheral capillaries (but may aggregate by becoming withdrawn from circulation when passing through the capillaries). Therefore, Hawking [6,8] proposed that malaria parasites burst to release their progeny (schizogony) at a specific time of day to coincide the maturation (which he termed ‘ripeness’) of gametocytes with the nocturnal foraging activity of their mosquito vectors. However, it is not clear whether Hawking envisaged transmission success to be maximized by daily rhythms in the maturation of gametocytes and/or density of mature gametocytes in the host's blood.

Tests of whether Hawking's hypothesis applies to malaria parasites have largely proved inconclusive [5]. Hawking [8] himself observed that mosquitoes feeding at night harboured more *Plasmodium knowlesi* oocysts. But Karunaweera [11] noted that mosquitoes fed at night time, during the height of *Plasmodium vivax* fever, produce less oocysts than those fed earlier in the day; no influence of time of day on oocyst burden has been reported for *Plasmodium chabaudi* [12] nor *Plasmodium falciparum* [13,14]; and gametocyte density can peak in the blood at the opposite time of day to when mosquitoes forage [15]. However, some of these studies did not contain many statistically independent replicates, with data originating from only 2 to 5 mice, single monkeys, 3 to 4 birds or 8 to 16 people per time point [8,12–16]. Despite the seeming lack of support, Hawking's hypothesis should not be prematurely rejected: a recent study of an avian malaria system reveals transmission to be most successful in the evening, although it is not clear how the parasites achieve this [16].

A difficulty with testing Hawking's hypothesis is that mosquitoes also display circadian rhythms in their physiologies, including immune responses [17,18]. When simply feeding mosquitoes at different times during the day, daily rhythms of both parasite and mosquito vary in an uncontrolled manner between the groups. Thus, the transmission benefits of parasite strategies deployed in the host could be undermined or exacerbated by mosquito rhythms. For instance, if rhythms exist in parasite transmissibility and mosquito susceptibility to infection, but their timing is inverted, then times of day when parasites are most transmissible are compensated for by reduced vector susceptibility. In this case, the net effect will disguise rhythms in both parasite transmissibility and mosquito susceptibility. Another problem can arise when studies only compare two time points of a single rhythm because they may coincidently sample when the rhythm reaches the same trait value as it ascends and descends (particularly if studying time points that are 12 h apart on a sine wave). Therefore, we examine periodicity in parasite transmission in the context of vector rhythms to understand the net outcomes and selective pressures on each party.

Understanding how rhythms in gametocyte infectivity interact with mosquito rhythms is urgently needed because mosquito populations are responding to the use of bed nets by shifting the time of day they forage for blood [19]. Here, unlike previous studies, we test the roles of daily rhythms in both parasite and vector and we examine parasite transmissibility from the host's blood throughout development in the vector. Specifically, we manipulate the time of day of blood feeding for both the mosquito vector *Anopheles stephensi* and the rodent malaria parasite *P. chabaudi*, quantify the densities of mature gametocytes in the blood at the time of feeding, as well as the intensities and prevalences of the resulting oocyst and sporozoite infections in the vector. We find that at night time, despite being less numerous in the blood, gametocytes are twice as infective to mosquitoes and that mosquitoes are more susceptible to infection during their rest phase in the day time. The enhanced infectiousness of gametocytes at night interacts with greater mosquito susceptibility in the daytime to elevate sporozoite burdens by fourfold.

## 2. Material and methods

### (a) Hosts, parasites and vectors

Vertebrate hosts were eight- to 11-week-old C57Bl/6 J female mice given access to food and drinking water (supplemented with 0.05% para-aminobenzoic acid [20]) ad libitum. We entrained 40 mice to a 12 L : 12 D h light : dark cycle three weeks prior to and during the experiment. At Zeitgeber time ZT3 (Zeitgeber time refers to hours since lights on, so ZT3 is 3 h after lights on), we infected all mice with 10^5^ rodent malaria *P. chabaudi* genotype AS (passage number A12) infected red blood cells at ring stage by intraperitoneal injection. We infected all experimental mice with parasites from donor mice entrained to the same photoperiod to avoid costs of mismatching the phase of parasite and host rhythms [21].

Mosquitoes were reared according to Spence *et al.* [22]. Briefly, we housed mosquitoes at 26°C, 70% relative humidity, 12 L : 12 D h cycle and provided them with 10% fructose solution, supplemented with 0.05% para-aminobenzoic acid. We randomly assigned *A. stephensi* mosquito pupae to three offset 12 L : 12 D h photoschedules according to their treatment groups (electronic supplementary material, figure S1). At 5 days post pupation, we randomly assigned female mosquitoes within each photoschedule to paper cages with meshed lids (20 replicate cages for each of the four experimental groups, with 60 mosquitoes per cage) and supplemented their sugar/para-aminobenzoic acid water with 0.05% gentamicin solution prior to blood feeding. We starved mosquitoes for exactly 24 h before blood feeding by providing them with access to water only. Blood feeds occurred when the mosquitoes were between 5 and 11 days post emergence, and mosquitoes were given sugar/para-aminobenzoic acid water immediately following blood feeding.

### (b) Perturbing time of day

**Figure 1.**
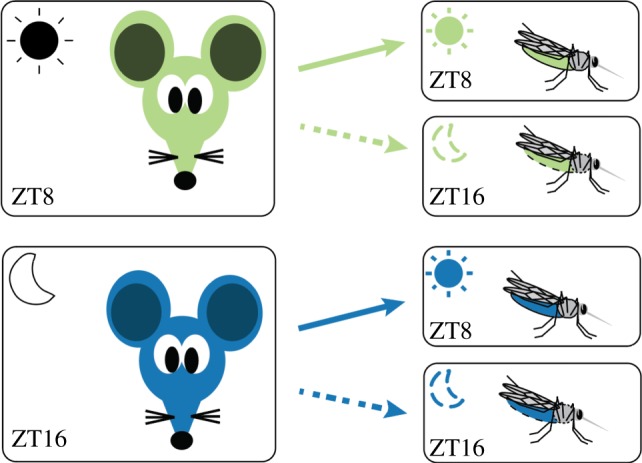
Experimental design. We infected 80 mice (over two blocks) with ring stages of *P. chabaudi* (genotype AS) at ZT3 to ensure parasite rhythms were in phase with the host's rhythms in all treatment groups [21,23]. At ZT8 (green), 40 mice were fed to 40 cages of mosquitoes experiencing their day (ZT8; solid arrow) or night (ZT16; dotted arrow) and we repeated this at ZT16 for the other 40 mice (blue). Gametocyte metrics were quantified in the two groups of mice just before exposure to mosquitoes and oocysts and sporozoites were followed in the four groups of mosquitoes. All feeds (i.e. at both ZT8 and ZT16) were performed in the dark to prevent unexpected light exposure to mosquitoes, which is known to affect biting behaviours and rhythms in gene expression [24,25].

Our experimental design involved four treatment groups in which mosquitoes fed at their ZT8 (mosquito daytime) or ZT16 (mosquito night time) on mice at their ZT8 (parasite daytime) or ZT16 (parasite night time) (figure 1). We chose these times of day for mosquitoes to minimize the risk of coincidentally sampling a rhythm when it reaches the same trait value as it ascends and descends. Specifically, ZT8 falls in the middle of the mosquitoes resting phase and at ZT16, they are several hours into their period of nocturnal activity (electronic supplementary material, figure S2). Blood stage *P. chabaudi* parasites have a 24 h rhythm in their cycles of asexual replication and are early in the developmental cycle (young trophozoites) at ZT8 and approaching maturation (schizogony) at ZT16, which is when gametocytes are produced [21]. The experiment was carried out in two blocks (‘1’ and ‘2’) in which *n* = 10 feeds per treatment group per block were initiated 2 days apart. Across both blocks, each treatment group comprised 20 mice, resulting in 80 mosquito feeds (80 cages of 60 mosquitoes per cage) and totalling 2400 mosquitoes.

We reared all experimental mice in the same photoschedule, with lights on at 1.00 GMT and off at 13.00 GMT. To cross factor ‘time zones’ for parasites and vectors, lights on and lights off occurred at different times with respect to GMT (though always with 12 L : 12 D h photoperiods) for the three offset mosquito photoschedules (electronic supplementary material, figure S1). Mosquito photoschedule 1 provided mosquitoes at their ZT16 to feed on ZT8 mice. Mosquito photoschedule 2 provided mosquitoes experiencing their ZT8 to feed on mice experiencing ZT8 as well as mosquitoes experiencing their ZT16 to feed on mice experiencing ZT16. Mosquito photoschedule 3 provided mosquitoes at their ZT8 to feed on mice experiencing ZT16. This design avoided the need to have multiple groups of mice, allowing all infections to be initiated from the same parasite stock and donor mice.

### (c) Transmissions and data collection

On day 14 post infection, at ZT8 or ZT16, we took blood samples from all mice by tail snip for quantification of gametocytes by RNA extraction [26] and subsequent quantitative reverse transcriptase PCR (RT-qPCR) targeting the sexual stage-specific expressed gene PCHAS_0620900, previously named PC302249.00.0 [27], which detects gametocytes from 30 to 35 h old onwards [26]. Immediately after sampling, we anaesthetised mice as per PPL 70/8546 using an injection of ketamine hydrochloride and medetomidine, and exposed each mouse to its cage of mosquitoes for 20 min and then euthanized them, following Spence *et al.* [22]. We carried out all mosquito feeds under dim red light to prevent differences in mosquito biting rates resulting from unexpected light exposure during blood feeding [24,25].

We quantified infection in mosquitoes at oocyst (day 8 post blood meal) and sporozoite (day 14 post blood meal) stages. For oocyst quantification, we dissected midguts from 20 cold anaesthetized mosquitoes per cage and visualized them microscopically after staining with 0.5% mercurochrome. To ensure that all mosquitoes in each experimental block could be processed within a single day, we took photographs of all midguts (electronic supplementary material, figure S3) and quantified oocysts using ImageJ software v. 1.51o (NIH, Bethesda, MD, USA). We quantified sporozoites from pools of five mosquitoes (*n* = 4 pools per cage) that were bisected between the second and third pair of legs following Foley *et al*. [28], ensuring that salivary gland sporozoites are quantified, not those remaining in oocysts. Mosquitoes were pooled to enable sufficient samples to be processed within a single day. The consequences of pooling are that we lose some statistical power in comparisons across treatment groups and the accuracy of estimates of sporozoite prevalence and density may be reduced. We extracted DNA using the CTAB protocol from Chen *et al.* [29] with minor modifications: tissue lysis was done by shaking a ball bearing at 30 bpm for 2 min in a tissue lysate machine; liquid volumes were increased; a chloroform step and 10 min chilled ethanol incubation were added and all centrifuge steps were performed at 4°C. We used qPCR targeting the 18S rRNA gene [30] to quantify the number of parasite genomes, assuming 1 genome per sporozoite. Sporozoite data for one mouse (mouse ZT16/mosquito ZT8) were lost owing to failed DNA extraction.

### (d) Additional experiments

We analysed an independently collected dataset to verify the observation of lower densities of gametocytes in host blood at night. This dataset originated from infected mice pre-treated with 125 mg kg^−1^ phenylhydrazine (PHZ) to induce anaemia and enhance gametocyte production. Specifically, 4 days after PHZ treatment, 4 eight-week-old C57Bl/6 J male mice were intravenously infected at ZT2 with 10^7^ *P. chabaudi* genotype CR-infected red blood cells at ring stage. On day 4 post infection, mature gametocyte densities were quantified at ZT5, 9, 17 and 21 by microscopy. This dataset was originally collected to assess exflagellation rates of male gametocytes, and microscopy (rather than PCR) is the best approach to detect exflagellation.

To investigate whether lower densities of gametocytes at night can be explained by gametocytes passively accumulating in the peripheral capillaries (e.g. by sequestration), we carried out an additional experiment. We used mosquitoes to sample blood from peripheral capillaries of mice and compared the density of gametocytes in their guts to those in the tail vein of the mice they fed on (three mice infected with 10^5^ *P. chabaudi* genotype ER-infected red blood cells at ring stage). On day 14 post infection, we made thin blood smears for gametocyte quantification from the tail vein in triplicate and immediately offered *A. stephensi* mosquitoes a blood meal following the same protocols as in the main experiment. We carried feeds out at ZT16 for both parasites and mosquitoes. Within 10 min post blood meal, we dissected five mosquitoes per feed and used the contents of their midguts to make thin blood smears. We stained all smears with Giemsa and quantified gametocytes by microscopy for the smears of tail vein blood (three per mouse) and mosquito midguts (five per mouse). Microscopy returns a more accurate estimate of gametocyte numbers than PCR because upon ingestion of a blood meal, male gametocytes rapidly undergo several rounds of mitosis. Because one mouse did not have any gametocytes, its data were excluded from analysis.

### (e) Statistical analysis

We performed all data analyses using R v. 3.2.4 (R Foundation for Statistical Computing, Vienna, Austria). Gametocyte densities were analysed using linear models, with a log_10_ transformation for gametocyte densities, while gametocyte densities, normalized to white blood cells, were compared between tail vein and mosquito midguts using linear mixed effect models with mouse fitted as a random effect to account for multiple samples taken from each mouse. We used linear mixed models to analyse oocyst and sporozoite densities, which were square root transformed to meet assumptions of normality and homogeneity of variance. Analogous analyses excluding uninfected mosquitoes retrieve similar patterns, leading to the same conclusions, and are presented in the electronic supplementary material, figure S4. We used generalized linear models, with a binomial error structure, to analyse: the proportion of fed mosquitoes; the proportion (prevalence) of oocyst-infected mosquitoes and the proportion of sporozoite-infected pools. Specifically, we analysed a two-vector binomial response variable including counts of fed/unfed or infected/uninfected mosquitoes (or pools of mosquitoes). We minimized models after comparison with log-likelihood ratio tests, for which test statistics and *p*-values are reported, to determine whether terms could be removed. The identity of each block was controlled for in all analyses presented in the main text, and the average effect sizes and s.e.m. are calculated across blocks. Briefly, block did not interact with any of the fixed effects fitted in any analyses and so, statistics for terms involving block are not presented in the main text. However, a breakdown of figures by block and full analysis results for interactions with block and main effects of block are presented in the electronic supplementary material, figures S5–S8. Note that overall sporozoite prevalence (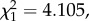 *p* = 0.043) and sporozoite burden (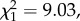 *p* = 0.003) differed between the blocks, but the patterns associated with parasite and mosquito times of day are statistically consistent across blocks. To illustrate the range of data and outliers, box and whisker plots of the data are shown in the electronic supplementary material, figure S9.

## 3. Results

### (a) Fewer gametocytes circulate at night

**Figure 2.**
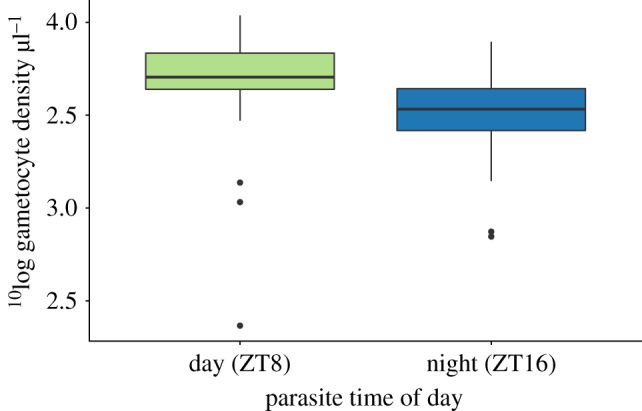
Gametocyte densities circulating in host blood are lower during the night time (ZT16) than the daytime (ZT8). *n* = 40 mice per time point.

Hawking's hypothesis would be supported if: (i) gametocytes circulate with higher densities at night; (ii) passively accumulate at night in the peripheral capillaries where they can be picked up by mosquitoes; or (iii) are more infectious at night. We first examined whether the densities of gametocytes circulating in host blood differs between day and night. Phenomena such as timed release from the bone marrow and age-specific mortality rates of gametocytes have been proposed to generate daily rhythms in the density of mature gametocytes [31]. We find that the densities of mature gametocytes are not higher at night time; instead, gametocyte densities are, on average, 1.6-fold (±s.e.m. 0.2) lower at night compared with the day (*F*_1,78_ = 11.11, *p* = 0.001) (figure 2; electronic supplementary material, figure S5). We confirmed this result in an independent experiment using a different *P. chabaudi* genotype (genotype CR), finding that gametocyte density is 1.5-fold (±s.e.m. 0.2) lower on average at night (ZT17-21) compared with the day (ZT5-9) (*F*_1,14_ = 5.70, *p* = 0.032).

Second, we examine whether the lower density of gametocytes observed at night is a consequence of time of day specific accumulation in the capillaries [12,32,33]. Blood samples to quantify gametocytes are taken from the host's tail vein, but mosquitoes harvest blood from subdermal capillaries in which mature gametocytes may passively accumulate by sequestration. Therefore, we carried out a further experiment to simultaneously compare gametocyte densities in blood from the host's tail vein with those in mosquito blood meals, at night (ZT16). When normalized to the density of white blood cells (following [33]), gametocyte densities varied across mice but are not significantly different in venous blood (0.62 ± s.e.m. 0.19 gametocytes white blood cell^−1^) and mosquito blood meals (0.57 ± s.e.m. 0.15 gametocytes white blood cell^−1^) (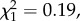 *p* = 0.659). Thus, the lower density of gametocytes in host blood at ZT16 is not explained by their accumulation in the peripheral circulation.

### (b) Mosquitoes are more likely to be infected from daytime blood meals

**Figure 3.**
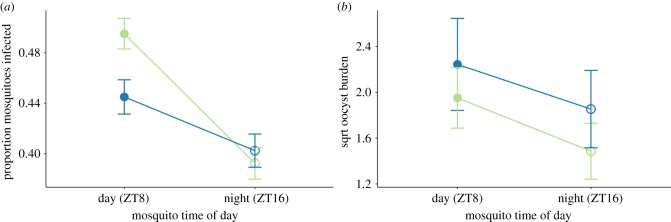
Night-fed mosquitoes are less likely to be infected (*a*) but oocyst burdens are not influenced by time of day for either party (*b*). Data presented are mean ± s.e.m. for the proportion of mosquitoes that are infected with oocysts (*a*) and oocyst burdens for all fed mosquitoes regardless of whether infected or not (*b*). Groups are: daytime (ZT8; closed symbols) and night time (ZT16; open symbols) feeding mosquitoes that fed on mice experiencing their day (ZT8; green) or night (ZT16; blue). Data in (*b*) are square root transformed to meet statistical model assumptions for analysis.

We observed no significant differences in the proportion of mosquitoes feeding (greater than 93% fed in all cages) with respect to the time of day of feeding for parasites (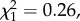 *p* = 0.608), mosquitoes (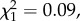 *p* = 0.766) or their interaction (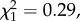 *p* = 0.590). Night-fed mosquitoes are 1.18-fold (±s.e.m. 0.05) less likely to harbour oocysts compared with mosquitoes that fed in their daytime (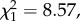 *p* = 0.003), irrespective of parasite time (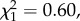 *p* = 0.437), or the interaction between mosquito and parasite time (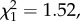 *p* = 0.218) (figure 3*a*; electronic supplementary material, figures 6A and 9A). However, oocyst burdens are not significantly influenced by time of day of feeding for parasites (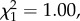 *p* = 0.318), mosquitoes (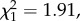 *p* = 0.167) or their interaction (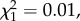 *p* = 0.931) (figure 3*b*; electronic supplementary material, figures S6B and S9B).

### (c) Gametocytes are more infectious at night

**Figure 4.**
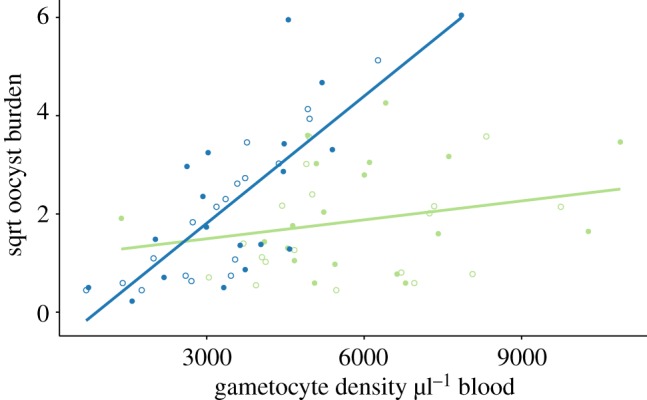
Gametocytes are more infective at night. Gametocytes taken up from hosts experiencing their night (ZT16; blue) are more likely to form oocysts than those taken up during the daytime (ZT8; green), regardless of time of day for mosquitoes (ZT8 closed and ZT16 open symbols). Gametocyte densities for each host are plotted against their corresponding mean oocyst burdens (square root transformed to meet model assumptions), and the fits are from linear regressions.

Fewer gametocytes are available to mosquitoes at night (figure 2), yet this reduced density does not affect the prevalence or intensity of oocysts. This suggests that gametocytes are more infectious at night. Infectivity can be assessed from the positive correlation between gametocyte density and oocyst burden; the steeper the slope the more likely a gametocyte is to develop into an oocyst. We find that time of day for mosquitoes (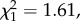 *p* = 0.204) and its interaction with parasite time of day (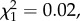 *p* = 0.889) do not significantly influence gametocyte infectiveness, but parasite time of day does (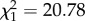, *p* < 0.001) (figure 4; electronic supplementary material, figure S7). Specifically, each gametocyte, on average, results in 2.22-fold (±s.e.m. 0.30) more oocysts when taken up at night compared with the daytime.

### (d) Onwards transmission is determined by time of day of both parasites and mosquitoes

**Figure 5.**
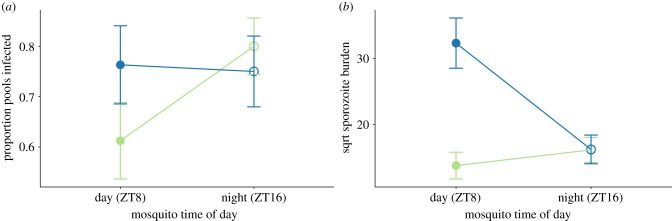
Parasite and mosquito time of day do not affect sporozoite prevalence (*a*) but do affect sporozoite burdens (*b*). Each sample consisted of a pool of five mosquitoes that blood fed on the same mouse (four samples per mouse): a positive pool requires that at least one of five mosquitoes were infected with sporozoites. Data presented are the mean ± s.e.m. for the proportion of sporozoite positive pools (*a*) and sporozoite burdens for all fed mosquitoes (*b*). Groups are: daytime (ZT8; closed symbols) and night time (ZT16; open symbols) feeding mosquitoes that fed on mice experiencing their day (ZT8; green) or night (ZT16; blue). Data in (*b*) are square root transformed to meet model assumptions.

Hawking focused on whether parasites have evolved to coordinate transmission activities in the host's blood with the blood-foraging activity of mosquito vectors. Yet, for such a strategy to be adaptive (i.e. maximize fitness), it should enhance the potential for transmission from vectors to new hosts. Only sporozoites in the salivary glands can be transmitted to new hosts, so both the probability of a mosquito harbouring sporozoites and sporozoite density determine the likelihood of onwards transmission. Sporozoite prevalence is 1.2-fold (±s.e.m. 0.13) lower in the second experimental block (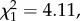 *p* = 0.043), but does not vary according to mosquito time of day (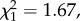 *p* = 0.196), parasite time of day (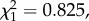 *p* = 0.364) or their interaction (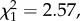 *p* = 0.109) (figure 5*a*; electronic supplementary material, figures S8A and S9C). Thus, sporozoite prevalence differs from the lower prevalence in night-fed mosquitoes that we observed for oocysts (figure 3*a*). Sporozoite burdens are also lower in the second block (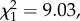 *p* = 0.003). However, in contrast to sporozoite prevalence, sporozoite burdens are shaped by an interaction between time of day for both parasites and mosquitoes (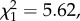 *p* = 0.018, figure 5*b*; electronic supplementary material, figures S8B and S9D). Across both experimental blocks, sporozoite burdens are 4.36-fold (± s.e.m. 1.17) higher in mosquitoes that fed during their daytime, but only when taking up gametocytes experiencing their night time (block 1: 3.20-fold ± s.e.m. 0.77; block 2: 7.30-fold ± s.e.m. 2.56).

## 4. Discussion

We have deployed modern techniques to quantify gametocytes and assess malaria transmission in an experiment with greater replication than previous studies to determine whether parasites can maximize transmission by coordinating their development with the foraging activity of the mosquito vector. We reveal that: (i) gametocytes are less numerous in the host's blood at night but night time gametocytes are more likely to develop to oocysts; (ii) the greater infectivity of night time gametocytes does not increase the probability that mosquitoes become infected or result in more intense infection at the oocyst stage; (iii) independently of parasite time of day, mosquitoes fed at night are less likely to be infected with oocysts; and (iv) the greater infectivity of night time gametocytes does not alter the probability of mosquitoes harbouring sporozoites but does increase sporozoite burden in mosquitoes fed in their daytime. Our findings are broadly consistent with a recent study on the avian malaria *Plasmodium relictum* [16], which also reveals that transmission (to oocyst stage) is more efficient in the late afternoon and night (when vectors are active) than at noon or early morning. However, in this study, enhanced transmission is associated with elevated parasite density in the avian host's blood in the late afternoon. Gametocytes appear to make up a large proportion of parasites in *P. relictum* infections, and because this is an asynchronous species of malaria parasites [34], it is possible that a late afternoon increase in gametocyte density explains enhanced transmission.

**Figure 6.**
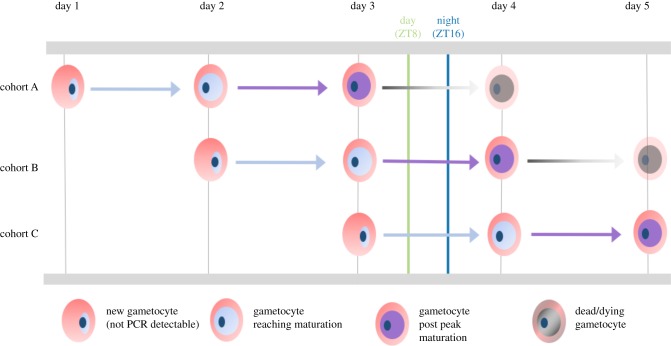
Dynamics of gametocyte development. A new cohort of gametocytes starts developing at every schizogony (approx. ZT17) and *P. chabaudi* gametocytes are thought to require approximately 36 h from schizogony to reach maturity (i.e. begin to mature at approx. ZT2 on second day) and remain mature (i.e. infective) for 12–18 h (i.e. up to ZT17–23 on the second day) before senescing [12,35]. The half-life of a mature gametocyte is approximately 14 h and most gametocytes are cleared by 60 h post schizogony (i.e. ZT12 on the third day [12,35]. During our experiment, we collected parasites at daytime (ZT8; green line) and night time (ZT16; blue line), thus sampling three cohorts of gametocytes (A–C), and our RT-qPCR assays detect gametocytes from 30 to 35 h post schizogony onwards, and thus, they are mature and only from cohorts A and B [26]. At ZT8, gametocytes comprised those produced on day 3 and not yet infective and not detected (cohort C); those produced on day 2 and reaching peak maturity (cohort B); and produced on day 1 and senescing (cohort A). Whereas, at ZT16, gametocytes were not yet infective and not detected (cohort C); at peak maturity (cohort B) and mostly cleared (cohort A). If more of cohort A's gametocytes are lost by ZT16, this could explain the lower density and the higher per-gametocyte infectivity observed at ZT16. Furthermore, if a cohort of gametocytes gradually attains maturity from approximately 36 h of age, more of cohort B's gametocytes will be infective at ZT16 than ZT8. The contributions of cycles of maturation, senescence and mortality may be further complicated by variable investment in gametocytes in sequential cohorts [36].

Finding that gametocytes of *P. chabaudi* are more infective at night provides partial support for Hawking's hypothesis. Having ruled out passive accumulation in the peripheral capillaries at night, several non-mutually exclusive explanations for a daily rhythm in infectivity remain. For the synchronous *P. chabaudi*, the developmental cycle of gametocytes offers a proximate (mechanistic) explanation (figure 6). Gametocytes have a finite lifespan and host immune responses can affect gametocyte infectivity, though their action on gametocytes circulating in the host is unclear [31]. Gametocytes quantified at ZT8 are the cohort (B, purple arrow) reaching sexual maturity plus the survivors of the previously produced cohort (A, grey arrow), whereas gametocytes quantified at ZT16 comprise mature gametocytes from cohort B (purple arrow) plus even fewer survivors from cohort A (grey arrow). Thus, the loss of gametocytes as the circadian cycle progresses is an unavoidable consequence of extrinsic mortality, but the attainment of maturity of the next cohort may compensate for the loss in numbers. Malaria parasite investment into gametocytes varies during infections [36] and so, differences in the numbers of gametocytes produced in sequential cohorts could affect the average age of a pool of gametocytes and exacerbate or erode a rhythm in infectivity.

Evolutionary (ultimate) explanations for timing gametocyte production (i.e. schizogony) according to figure 6 may include maximizing the proportion of gametocytes that are mature at the time of transmission. This metric will be under stronger selection than the density of mature gametocytes if immature or senesced gametocytes interfere with mating success in the blood meal. For instance, the time available to mate in the blood meal is short and males are often limiting [35,37,38], and so, attempts to fertilize senesced females may ‘waste’ males [39]. In this scenario, parasite evolution is shaped by processes operating in the vector. In addition, gametocyte timing might be selected for by within-host processes. For example, host immune responses that reduce gametocyte infectivity may be upregulated at night and so, parasites must compensate for this by maximizing infectivity at night. This assumes that there are costs of being maximally infective throughout the circadian cycle and/or that gametocytes only have a short duration of maximal infectivity owing to constraints on the processes involved in development and/or senescence. Alternatively, the timing of schizogony in *P. chabaudi* may be solely determined by the time of day that hosts feed because parasites must coordinate the development of asexual stages with resources such as glucose [40,41]. Prioritizing the timing of asexual development thus constrains when gametocytes are produced and imposes the trade-off between the density and infectivity.

Our data reveal that mosquitoes fed at night are less susceptible to infection and that vector rhythms have long-lasting effects on sporogony. Similarly, long-lasting effects of time of day of infection are observed for the expulsion rate of the intestinal parasitic helminth, *Trichuris muris* several weeks after infection [42]. Why might mosquitoes fed at night be less susceptible to infection? Rhythmic immune defences have been described in insects [43,44], including *Anopheline* mosquitoes [17]. For example, sporozoites are phagocytosed by haemocytes in mosquitoes [45] and the protective capacity of phagocytic cells peaks at night in *Drosophila* [43]. Furthermore, transcriptional profiling in *Anopheles gambiae* has revealed that at least 20 genes of known or putative immune function (including against malaria parasites) display diel expression patterns with variable timing (phases) [18]. In addition to immune responses, daily rhythms in physiologies involved in dealing with blood digestion, the osmotic challenge of a blood meal, and in gut microbe communities could all be involved in mosquito susceptibility. That previous studies have not recognized the potential for time of day to affect the outcome of interactions between malaria parasite rhythms and vector rhythms, nor followed parasites throughout sporogony, may explain their lack of support for Hawking's hypothesis.

Given that mosquito populations are responding to the use of bed nets by shifting the time of day they forage for blood [19], understanding how time of day shapes interactions between parasites and mosquitoes is necessary to predict the epidemiological consequences of altered mosquito rhythms [46]. Clearly, if bed nets prevent night time transmission, then day-biting is beneficial for parasites, but if human malaria parasites behave as we observe for *P. chabaudi*, transmission potential may not change (i.e. mosquitoes fed at ZT8 on ZT8 parasites harbour the same sporozoite burdens as mosquitoes fed at ZT16 on ZT16 parasites). By contrast, if parasites can alter their rhythms, they may be able to capitalize on the greater transmission potential of (currently) night time gametocytes that infect mosquitoes feeding in the day. However, the fitness consequences of coordinating the development of asexually replicating stages with the host's circadian rhythms [21,23,41] may constrain the capacity of parasites to adjust the timing of schizogony. In this case, the duration of gametocyte development may be selected on, necessitating investigations into the flexibility of gametocyte developmental duration. Furthermore, given that mosquitoes will be under selection to cope with blood feeding in the daytime by changing the timing of associated physiological processes [46], the overall impact on malaria transmission is hard to predict.

The notion that rhythms in transmission traits expressed by parasites maximize onwards transmission has also been applied to parasite species that do not require a vector. For example, peak shedding of *Isospora* sp. from its avian host occurs in the late afternoon and this timing is thought to minimize damage from UV radiation while the parasite waits to encounter a new host [47]. The cercariae of *Schistosoma* are proposed to time their emergence from the intermediate snail host in the morning or afternoon according to whether they specialize on livestock or nocturnal rodents for their final host [47–50]. Finding support for Hawking's hypothesis in malaria parasites should motivate tests of whether periodicity in transmission behaviours maximizes fitness for other parasite lifestyles.

## Supplementary Material

SI figure 1-9

## Data accessibility

The data supporting the findings in this study are deposited in the Dryad Digital Repository: http://dx.doi.org/10.5061/dryad.g0j11kf [51].
