# Supplementary material for: Adaptive periodicity in the infectivity of malaria gametocytes to mosquitoes
Source: Proc Biol Sci. 2018 Oct 3;285(1888):20181876. doi: 10.1098/rspb.2018.1876 (PMC6191691; doi:10.1098/rspb.2018.1876)
Supplement: SI figure 1-9 [file rspb20181876supp1.pdf]

Adaptive periodicity in the infectivity of malaria gametocytes to mosquitoes

Supporting information

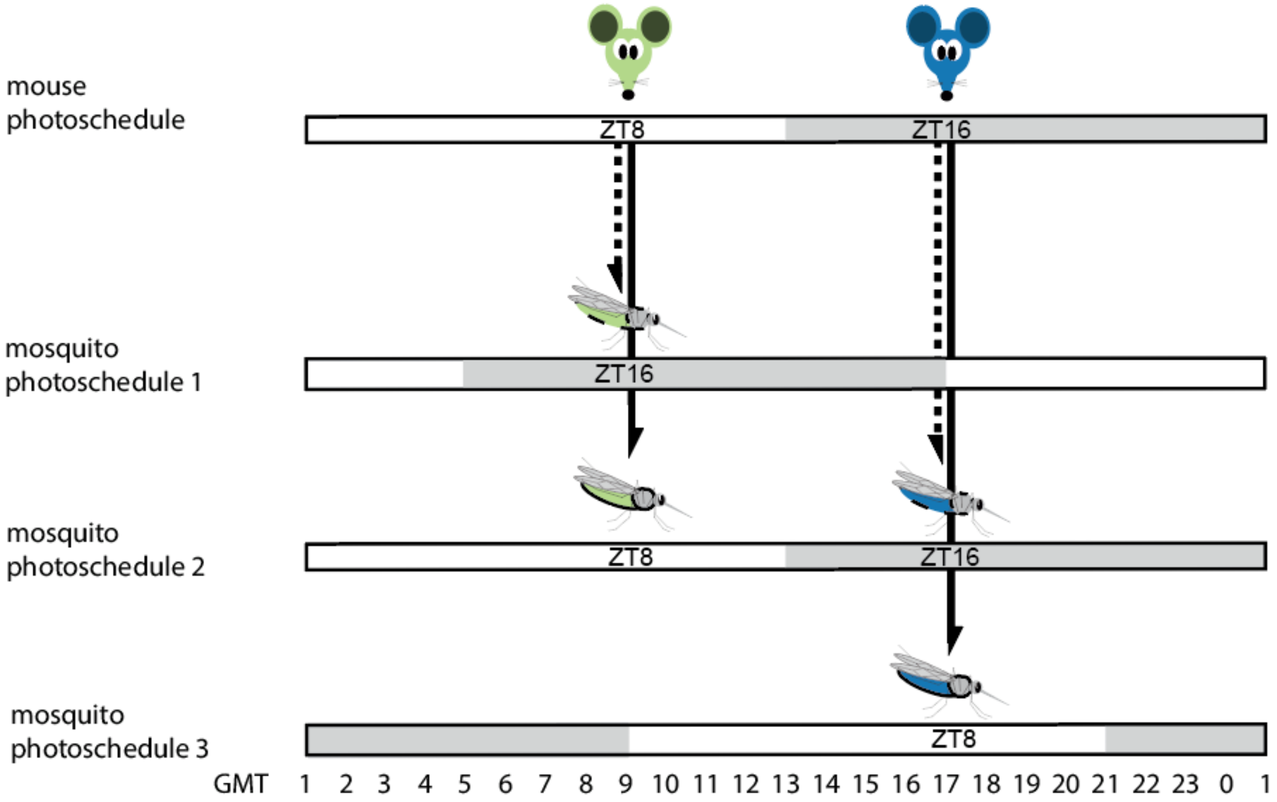

SI Figure 1. Offset photoschedules used in our experiment. We reared all experimental mice in the same photoschedule, with lights on at 1:00 GMT and off at 13:00 GMT. To cross factor “time zones” for parasites and vectors we entrained mosquitoes to three offset photoschedules where lights on and lights off occurred at different times with respect to GMT (though always in a 12:12 hrs light:dark cycle). Mosquito photoschedule 1 provided mosquitoes at their ZT16 to feed on ZT8 mice (green, dotted arrow). Mosquito photoschedule 2 provided mosquitoes experiencing their ZT8 to feed on mice experiencing ZT8 (green, solid arrow) as well as mosquitoes experiencing their ZT16 to feed on mice experiencing ZT16 (blue, dotted arrow). Mosquito photoschedule 3 provided mosquitoes at their ZT8 to feed on mice experiencing ZT16 (blue, solid arrow).

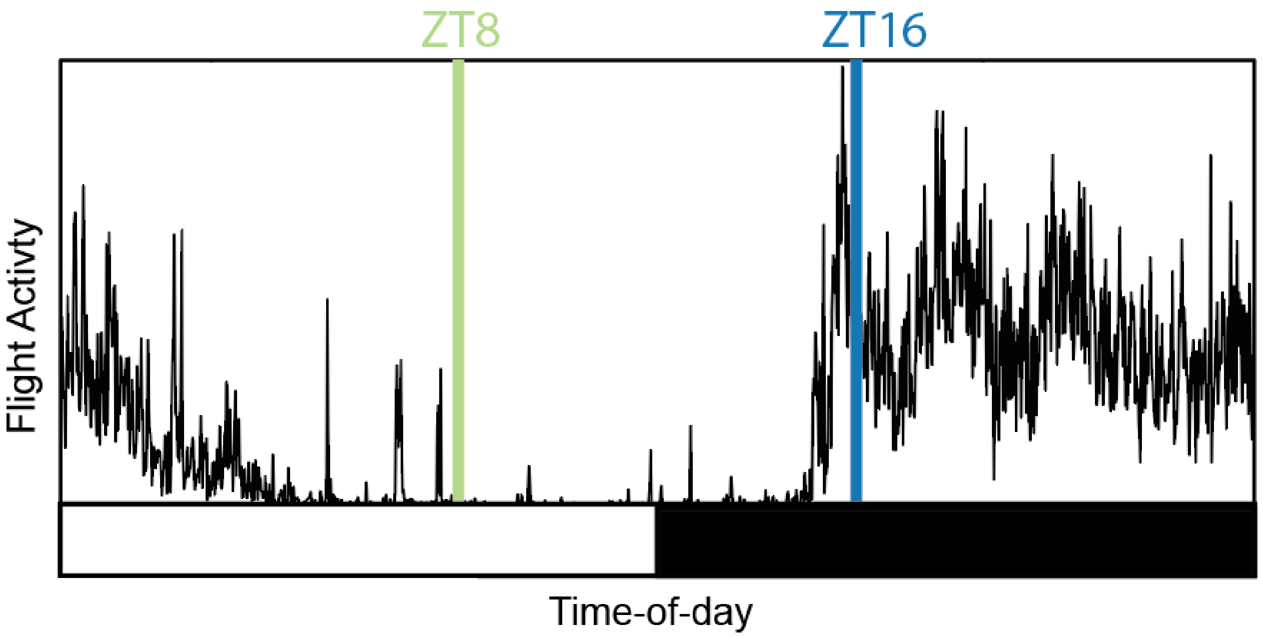

SI Figure 2. *An. stephensi* display daily rhythms in flight activity. White and black bars indicate lights on and off. Shown is the average 24-hr activity profile averaged over 3 days of activity for 32 females. Locomotor Activity Monitor units (LAM 25) (TriKinetics, Waltham, MA, USA) record individual mosquito locomotor flight activity and we processed data using ClockLab software (Actimetrics, Wilmette IL). Recordings were performed on female mosquitoes from the same cohort as the experimental mosquitoes used in this study and began on the day we gave blood meals to the experimental mosquitoes at the times indicated by the green and blue lines.

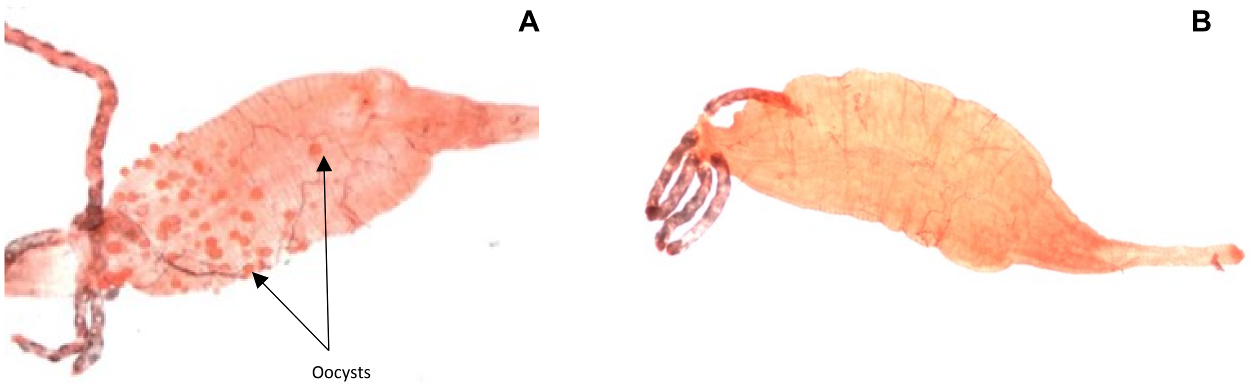

SI Figure 3. Example midguts with (A) and without (B) oocysts.

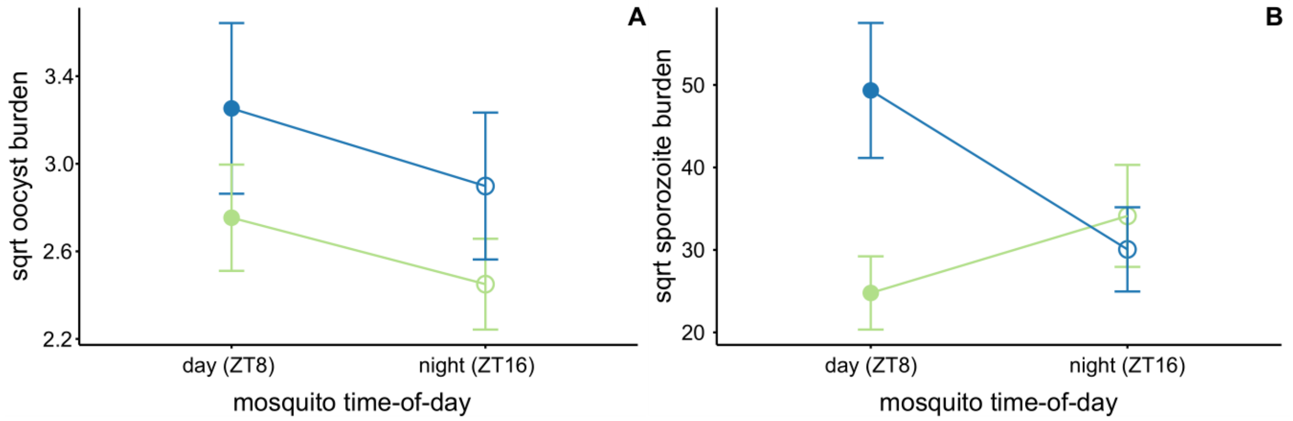

OocDens~ ParTime*MosqTime*Block

ParTime:MosqTime:Block	χ^2_1 =0.10, *P*=0.812
ParTime:MosqTime	χ^2_1 =0.01, *P*=0.928
MosqTime:Block	χ^2_1 =0.16, *P*=0.761
ParTime:Block	χ^2_1 =0.19, *P*=0.741
Block	χ^2_1 =0.05, *P*=0.862
MosqTime	χ^2_1 =2.00, *P*=0.276
ParTime	χ^2_1 =4.16, *P*=0.117

SporDens~ ParTime*MosqTime*Block

ParTime:MosqTime:Block	χ^2_1 =0.99, *P*=0.320
MosqTime:Block	χ^2_1 =2.02, *P*=0.156
ParTime:Block	χ^2_1 =2.55, *P*=0.110
ParTime:MosqTime	χ^2_1 =4.02, *P*=0.045
Block	χ^2_1 =7.43, *P*=0.006

SI Fig 4. Oocyst burdens of individual infected mosquitoes are not influenced by time-of-day for parasites or the mosquitoes (A). Parasite and mosquito time-of-day do affect sporozoite burdens of infected pools of mosquitoes (B). Each sample in B consisted of a pool of 5 mosquitoes that blood fed on the same mouse (4 samples per mouse): a positive pool requires that at least 1 of 5 mosquitoes were infected with sporozoites. Data presented are mean ± SEM burdens. Groups are: daytime (ZT8; closed symbols) and night time (ZT16; open symbols) feeding mosquitoes that fed on mice experiencing their day (ZT8; green) or night (ZT16; blue). Results of the statistical analyses including nonsignificant variables removed from each model are presented below each figure. Oocyst and sporozoite burdens were analysed by linear mixed models, with densities square root transformed to meet model assumptions. Mouse was used as a random effect (multiple (pools of) mosquitoes fed on each mouse). ParTime: parasite time, ZT8 or ZT16; MosqTime: mosquito time, ZT8 or ZT16.

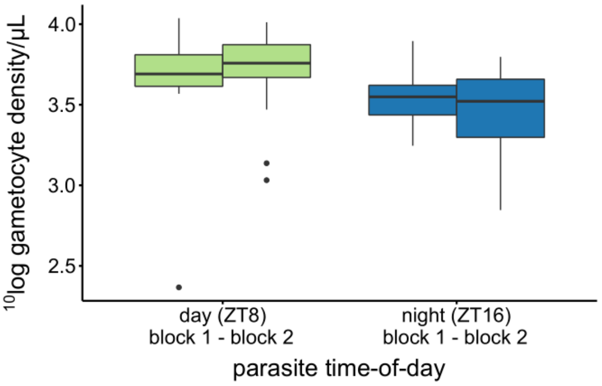

Model: GctDens ~ ParTime*MosqTime*Block

ParTime:MosqTime:Block	$F_{1,72}$=2.60; *P*=0.111
ParTime:MosqTime	$F_{1,73}$=0.01; *P*=0.933
MosqTime:Block	$F_{1,74}$=1.16; *P*=0.286
ParTime:Block	$F_{1,75}$=1.25; *P*=0.267
Block	$F_{1,76}$=0.03; *P*=0.865
MosqTime	$F_{1,77}$=0.76; *P*=0.386
ParTime	$F_{1,78}$=11.1; *P*=0.001

SI Figure 5. Gametocyte densities circulating in host blood are lower during the night time (ZT16) than the daytime (ZT8) in both experimental blocks. N=20 mice per group. The data and analysis are summarised in Fig. 2 in the main text but split into blocks 1 and 2 to illustrate between-repeat variability, and results of the statistical analysis including nonsignificant variables removed from the model. Gametocyte densities were analysed with linear models, with gametocyte densities ^{10}log transformed to meet model assumptions. GctDens: gametocyte density; ParTime: parasite time, ZT8 or ZT16; MosqTime: mosquito time, ZT8 or ZT16.

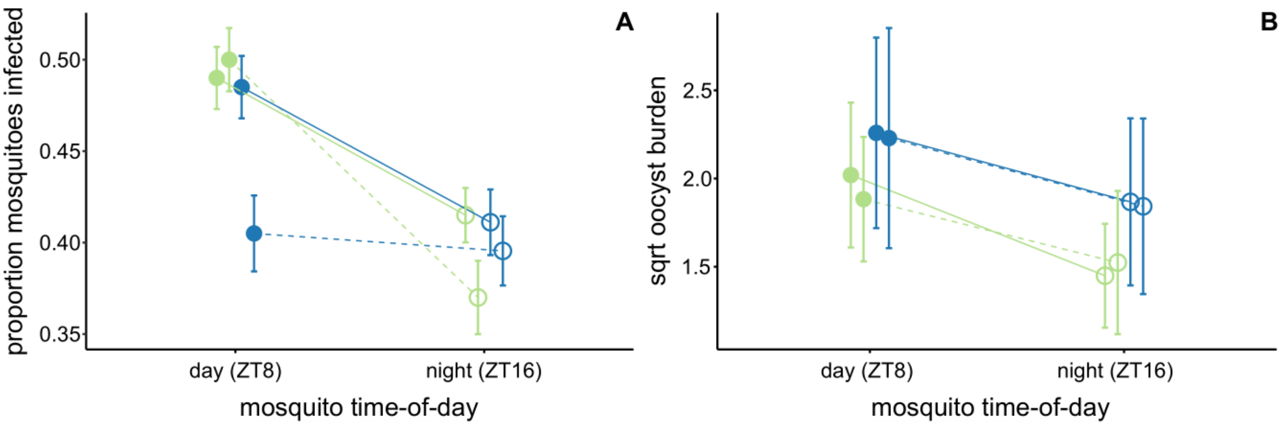

(Inf, Uninf)~ParTime*MosqTime*Block

ParTime:MosqTime:Block	χ^2_1 =1.45, P=0.229
MosqTime:Block	χ^2_1 =0.01, P=0.943
ParTime:Block	χ^2_1 =0.38, P=0.537
ParTime:MosqTime	χ^2_1 =1.52, P=0.218
ParTime	χ^2_1 =0.60, P=0.437
Block	χ^2_1 =1.72, P=0.190
MosqTime	χ^2_1 =8.57, P=0.003

OocDens~ ParTime*MosqTime*Block

ParTime:MosqTime:Block	χ^2_1 =0.01, P=0.943
ParTime:MosqTime	χ^2_1 =0.01, P=0.931
ParTime:Block	χ^2_1 =0.01, P=0.925
MosqTime:Block	χ^2_1 =0.05, P=0.818
Block	χ^2_1 =0.01, P=0.923
ParTime	χ^2_1 =1.00, P=0.318
MosqTime	χ^2_1 =1.91, P=0.167

SI Figure 6. Night fed mosquitoes are less likely to be infected (A) but oocyst burdens are not influenced by time-of-day for mosquitoes or parasites (B). Data presented are mean ± SEM over mice in each group, for the proportion of mosquitoes that are infected with oocysts (A) and oocyst burdens for all fed mosquitoes regardless of infection status (B). The data and analysis are summarised in Fig. 3 in the main text but split into blocks 1 (solid lines) and 2 (dashed lines) to reveal between-repeat variability. Groups consist of daytime (ZT8; closed symbols) and night time (ZT16; open symbols) feeding mosquitoes that fed on mice experiencing their day (ZT8; green) or night (ZT16; blue). Results of the statistical analyses including nonsignificant variables removed from each model are presented below each figure. Oocyst prevalences were analysed by binomial generalised linear models using the numbers of oocyst-infected and oocyst-uninfected mosquitoes as a two-vector variable (Inf,Uninf). Oocyst burdens were analysed by linear mixed models, with oocyst densities square root transformed to meet model assumptions. Mouse was used as a random effect (multiple mosquitoes fed on each mouse) ParTime: parasite time, ZT8 or ZT16; MosqTime: mosquito time, ZT8 or ZT16.

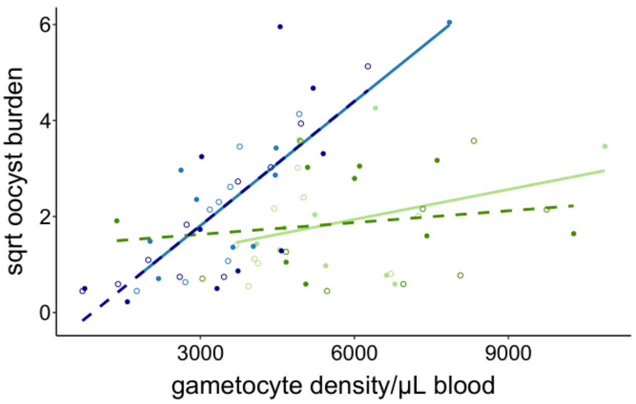

Ooc/gct~ ParTime*MosqTime*Block

ParTime:MosqTime:Block	χ^2_1 =0.02, *P*=0.876
ParTime:MosqTime	χ^2_1 =0.02, *P*=0.889
ParTime:Block	χ^2_1 =0.03, *P*=0.860
MosqTime:Block	χ^2_1 =0.06, *P*=0.801
Block	χ^2_1 =0.29, *P*=0.590
MosqTime	χ^2_1 =1.61, *P*=0.204
ParTime	χ^2_1 =20.8, *P*<0.001

SI Figure 7. Gametocytes are more infective at night. Gametocytes taken up from hosts experiencing their night (ZT16; blue) are more likely to form oocysts than those taken up during the daytime (ZT8; green), regardless of time-of-day for mosquitoes (ZT8 closed, ZT16 open symbols). The data and analysis are summarised in Fig. 4 in the main text but split into blocks 1 (light symbols, solid lines) and 2 (dark symbols, dashed lines) blocks to reveal between-repeat variability. Gametocyte densities for each host are plotted against their corresponding mean oocyst burdens (square root transformed to meet model assumptions), and the fits are from linear regressions. Note the fits for night time (ZT16) gametocytes in both blocks are identical. Results of the statistical analyses including nonsignificant variables removed from the model are presented below each figure. The ratio of oocysts to gametocytes was analysed by linear mixed models, using mouse as a random effect (multiple mosquitoes fed on each mouse) with oocyst ratios square root transformed to meet model assumptions. ParTime: parasite time, ZT8 or ZT16; MosqTime: mosquito time, ZT8 or ZT16.

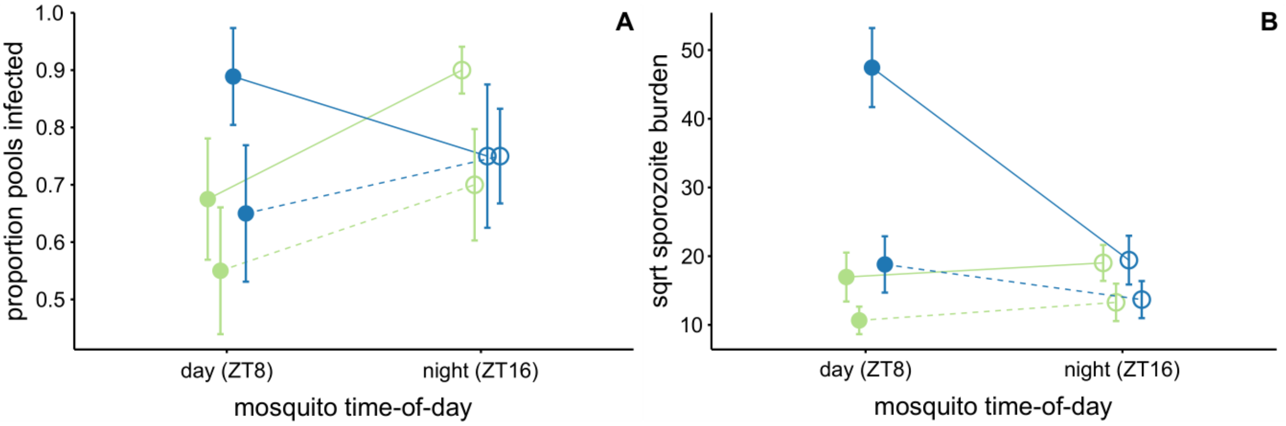

**(Inf, Uninf)~ParTime*MosqTime*Block**	
ParTime:MosqTime:Block	χ^2_1 =2.355, *P*=0.125
ParTime:Block	χ^2_1 =0.004, *P*=0.946
MosqTime:Block	χ^2_1 =0.197, *P*=0.657
ParTime:MosqTime	χ^2_1 =2.573, *P*=0.109
ParTime	χ^2_1 =0.825, *P*=0.364
MosqTime	χ^2_1 =1.669, *P*=0.196
Block	χ^2_1 =4.105, *P*=0.043

**SporDens~ ParTime*MosqTime*Block**	
ParTime:MosqTime:Block	χ^2_1 =2.36, *P*=0.125
ParTime:Block	χ^2_1 =2.18, *P*=0.139
MosqTime:Block	χ^2_1 =2.39, *P*=0.122
ParTime:MosqTime	χ^2_1 =5.61, *P*=0.018
Block	χ^2_1 =9.03, *P*=0.003

SI Figure 8. Parasite and mosquito time-of-day do not affect sporozoite prevalence (A) but do affect sporozoite burdens (B). Each sample consisted of a pool of 5 mosquitoes that blood fed on the same mouse (4 samples per mouse): a positive pool requires that at least 1 of 5 mosquitoes were infected with sporozoites. Data presented are the mean ± SEM over mice in each group, for the proportion of sporozoite positive pools (A) and sporozoite burdens for all fed mosquitoes regardless of infection status (B). The data and analysis are summarised in Fig. 5 in the main text but split into blocks 1 (solid lines) and 2 (dashed lines) blocks to reveal between-repeat variability. Groups are: daytime (ZT8; closed symbols) and night time (ZT16; open symbols) feeding mosquitoes that fed on mice experiencing their day (ZT8; green) or night (ZT16; blue). Data in B are square root transformed to meet model assumptions. Results of the statistical analyses including nonsignificant variables removed from the model are presented below each figure. Sporozoite prevalences were analysed by binomial generalised linear models using the numbers of sporozoite-infected and sporozoite-uninfected pools as a two-vector variable (Inf,Uninf). Sporozoite burdens were analysed by linear mixed models with sporozoite densities square root transformed to meet model assumptions. Mouse was fitted as a random effect (multiple pools of mosquitoes fed on each mouse). ParTime: parasite time, ZT8 or ZT16; MosqTime: mosquito time, ZT8 or ZT16.

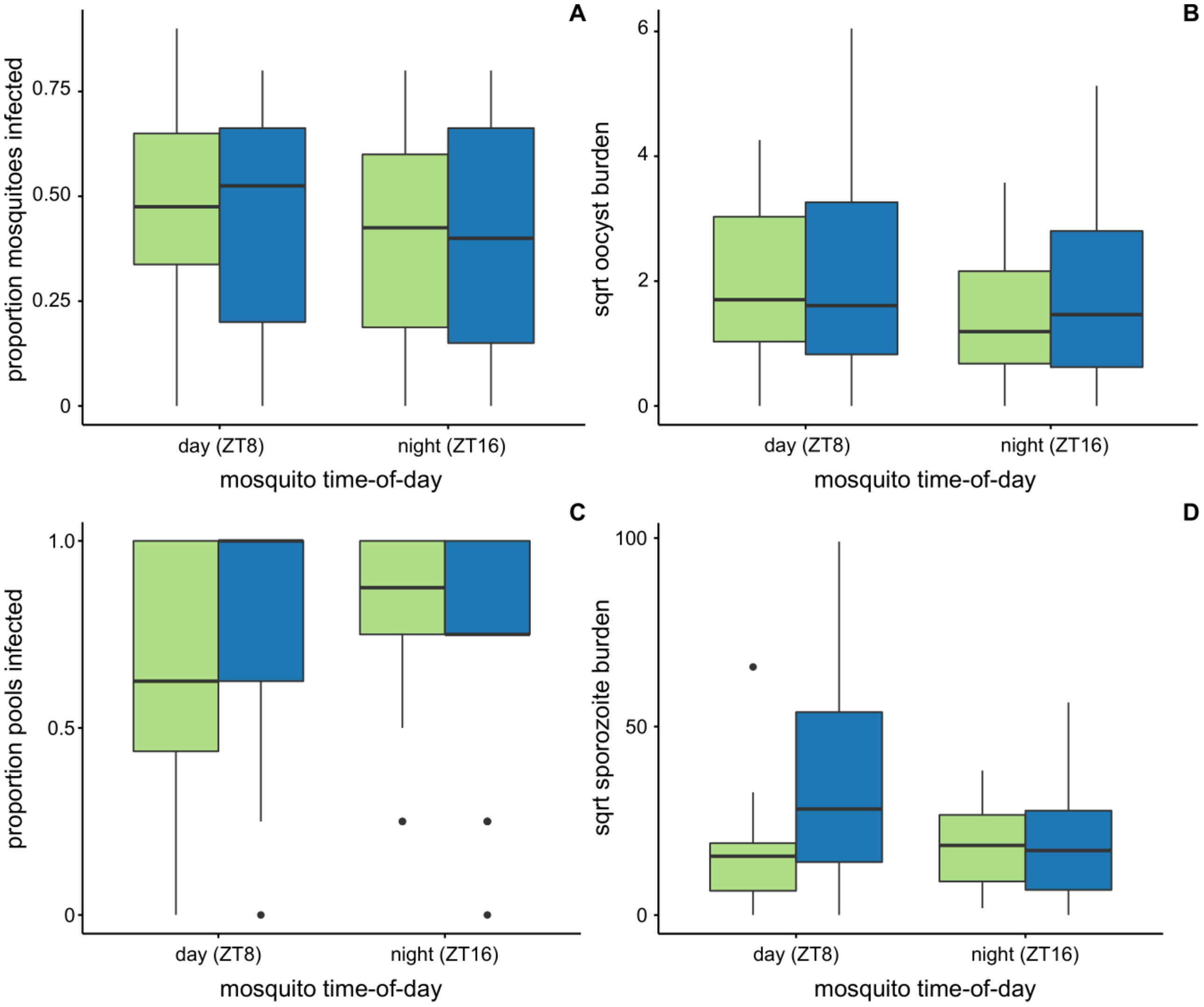

SI Figure 9. Box and whisker plot for oocyst prevalence (A), oocyst burden (B), sporozoite prevalence (C), and sporozoite burden (D) of all fed mosquitoes. Medians are shown, with the boxes illustrating 25-75 percentiles. Whiskers are Tukey style and outliers are plotted as dots. Groups consist of daytime (ZT8) and night time (ZT16) feeding mosquitoes that fed on mice experiencing their day (ZT8; green) or night (ZT16; blue).
